# Culicidae Fauna (Diptera: Culicomorpha) of the Quilombola Community of Abacatal, Ananindeua, Pará, in the Brazilian Amazon

**DOI:** 10.3390/insects16040397

**Published:** 2025-04-10

**Authors:** Hanna Carolina Farias Reis, Daniel Damous Dias, Bruna Laís Sena do Nascimento, Lucia Aline Moura Reis, Lucas Henrique da Silva e Silva, Fábio Silva da Silva, Durval Bertram Rodrigues Vieira, Roberto Carlos Feitosa Brandão, Eliana Vieira Pinto da Silva, Joaquim Pinto Nunes Neto

**Affiliations:** 1 Graduate Program in Parasitic Biology in the Amazon, Center for Biological and Health Sciences, State University of Pará, Belém 66095-663, Brazil; h.carolinafreis@gmail.com (H.C.F.R.); damous1994@gmail.com (D.D.D.); lucashenriqueuepa@gmail.com (L.H.d.S.e.S.); fabiosilva_micro@hotmail.com (F.S.d.S.); 2 Department of Arbovirology and Hemorrhagic Fevers, Evandro Chagas Institute, Secretariat of Health and Environment Surveillance, Ministry of Health, Ananindeua 67030-000, PA, Brazil; brunanascimento@iec.gov.br (B.L.S.d.N.); luciaalinereis@gmail.com (L.A.M.R.); durvalvieira@iec.gov.br (D.B.R.V.); robertobrandao@iec.gov.br (R.C.F.B.); elianapinto@iec.gov.br (E.V.P.d.S.)

**Keywords:** Quilombola, mosquito biodiversity, monitoring, entomological surveillance

## Abstract

The Quilombola community of Abacatal, located in Ananindeua, in the state of Pará, has characteristics that favor the proliferation of mosquitoes. Faunal surveys in environmental preservation areas are essential for understanding the dynamics of these vectors, whose epidemiological implications are significant. Uncontrolled human activities have an impact on temperature, humidity, and rainfall. The aim of this study was to survey the diversity of mosquito species in the Quilombola community of Abacatal. Field collections were carried out over 10 days, during the rainy and dry seasons, using the following methods: protected human attraction (PHA) and CDC traps. The results of the taxonomic identification of the samples collected revealed that the species *Coquillettidia* (*Rhynchotaenia*) *venezuelensis* and *Culex* (*Melanoconion*) *portesi* were eudominant in the area studied. The identification of species of epidemiological importance, which act as vectors for various arboviruses, highlights the relevance of monitoring in the area, especially considering that it will undergo a process of anthropization.

## Simple Summary

The Amazon region, renowned for its vast biodiversity, has suffered from environmental degradation, mainly due to deforestation and urbanization. These changes directly impact the region’s climate, influencing mosquitoes of the Culicidae family, which are vectors of major arthropod-borne diseases such as malaria and filariasis, and arboviruses, including *Orthoflavivirus denguei*, *Orthoflavivirus flavi*, *Orthoflavivirus nilense*, *Orthoflavivirus zikaense*, and *Alphavirus chikungunya*. The aim of this study was to investigate the diversity of Culicidae in a forest fragment in the metropolitan region of Belém. Two excursions were carried out in 2023. The collection methods used were human attraction and CDC traps, in the ground and using canopy modalities. A total of 3202 specimens were collected, classified into 56 species and 13 genera. Among the genera collected, 45 species were identified during the rainy season and 27 species during the dry season. The genus *Culex* exhibited the highest abundance, followed by the genus *Coquillettidia*. The recording of species that are involved in the transmission cycle of parasitic diseases and arboviruses in the Quilombola community of Abacatal shows us the need to monitor these vector populations as well as events of epizootics and febrile outbreaks in the human population living in this environment.

## 1. Introduction

The Amazon region is renowned for its vast biodiversity, covering approximately 45% of Brazil’s national territory. However, uncontrolled degradation, primarily driven by deforestation, has caused significant environmental impacts, altering local temperature, humidity, and rainfall patterns. These changes have led to shifts in species distribution, with many migrating to urbanized areas. In preserved environments, mosquito populations tend to be more diverse, but urbanization can significantly alter the dynamics of these vectors [1,2].

Understanding the diversity, spatial distribution, and bionomics of Culicidae Meigen, 1818 is essential for epidemiological studies, as many species in this group act as vectors for infectious and parasitic agents, including arboviruses. Moreover, mosquitoes can serve as indicators of environmental quality, as their presence or absence reflects ecosystem degradation or preservation. Variations in mosquito community composition are closely linked to temperature and rainfall, factors that directly influence their biological cycles. However, human activities have intensified environmental disturbances, favoring the proliferation of certain species over others [3,4].

As early as 1905, Emílio Goeldi recognized the importance of studying the Amazonian entomofauna in his book Mosquitos do Pará. His work highlighted the region’s rich biodiversity and the need for ecosystem conservation, and provided a detailed classification of mosquito species in Pará, describing their morphology, habits, and ecology [5]. Decades later, Kumm and Novis (1938) [6] conducted a survey on Marajó Island, focusing on sylvatic yellow fever. Their study expanded knowledge on mosquito distribution in the state, recording 80 species and providing one of the first faunal inventories for the region. In 1961, Cerqueira conducted a pioneering study on the distribution of Culicidae in the Amazon, analyzing samples from the now-extinct National Yellow Fever Service and collections from the National Institute for Amazonian Research. He recorded a total of 218 species across the Amazon region, of which 152 were documented in 57 localities within Pará. This study was a milestone in understanding the diversity and distribution of mosquitoes both at the regional and state levels. Subsequently, Xavier and Mattos (1975) [7] conducted one of the most extensive surveys on Culicidae in Pará. Sampling 307 sites across 64 municipalities, they identified 207 species, significantly expanding knowledge of mosquito diversity in the state. Since this study, new records on mosquito fauna and distribution have primarily emerged from faunal inventories and entomovirological investigations [8,9,10,11,12,13,14,15,16,17,18,19].

This extensive history of research underscores the Amazon’s critical role as a hotspot for arthropod-borne diseases, particularly arboviruses. The region is one of the largest ecosystems for such diseases, with 220 species identified, 37 of which are known to infect humans [20]. Among the main vectors, mosquitoes play a key role in arbovirus transmission and thrive in the Amazon region, where environmental conditions such as high humidity, abundant rainfall, and dense vegetation create an ideal habitat for their proliferation. These factors, combined with the adaptability of mosquitoes, sustain viral cycles and contribute to the recurrence of arboviral outbreaks, posing significant public health challenges. Notably, urban centers in the Amazon have been frequent epicenters of epidemics, highlighting the complex interaction between vector biology, environmental factors, and human activity [21,22].

Among the most relevant arboviruses for human health in the Amazon region are *Orthoflavivirus denguei*, *Orthoflavivirus flavi*, *Alphavirus mayaro*, and *Oropouche orthobunyavirus*. Each of these viruses presents distinct clinical characteristics, vectors, and significant public health impacts, particularly in a region of high biodiversity such as the Amazon [20]. *Orthoflavivirus denguei*, primarily transmitted by the mosquito *Aedes aegypti*, has caused recurrent outbreaks in Brazil since its reintroduction in the 1980s, with particularly severe impacts in urban areas [22]. *Orthoflavivirus flavi* (Yellow fever virus), which maintains an enzootic cycle in the Amazon region, is transmitted by mosquitoes of the genera *Haemagogus* Williston, 1896 and *Sabethes* Robineau-Desvoidy, 1827 [23]. This virus is responsible for periodic outbreaks, with human cases often associated with epizootics in non-human primates [24]. *Alphavirus mayaro*, also transmitted by mosquitoes of the genus *Haemagogus*, circulates mainly in rural and sylvatic areas but has the potential to cause outbreaks in human populations, especially in forested regions [24]. The *Oropouche orthobunyavirus*, originally identified in 1960, reemerged in 2024 with a significant outbreak in Brazil, totaling 10,940 confirmed cases in 22 of the country’s 27 states. The Amazon region was the most affected, accounting for 52.9% of the cases [25]. However, the occurrence of infections in non-Amazonian states highlights the virus’s ability to spread beyond its traditional endemic area. Although *Culicoides paraensis* (family Ceratopogonidae) is recognized as its primary vector, Culicidae species such as *Aedes serratus*, *Coquillettidia venezuelensis*, and *Culex quinquefasciatus* have been implicated in secondary transmission, increasing the virus’s dispersal potential and impact in rural and peri-urban areas [26].

The recent introduction of exotic arboviruses, such as *Alphavirus chikungunya* and *Orthoflavivirus zikaense*, has posed new challenges to public health in the Amazon. Both viruses have demonstrated rapid spread and adaptation to the Amazonian environment, with significant outbreaks reported in both urban and rural areas. These arboviruses are primarily transmitted by *Aedes aegypti* [27,28]. The coexistence of these viruses with native arboviruses raises questions about competition between them and their impact on transmission dynamics, particularly considering their ability to adapt to different ecosystems [27]. The Amazon, with its unique biodiversity and favorable ecological conditions, remains a critical setting for the emergence and reemergence of arboviruses, requiring constant surveillance and integrated control strategies.

Understanding the ecological factors influencing mosquito populations is essential for anticipating epidemiological risks in this rapidly changing landscape. In this context, the present study surveyed the Culicidae fauna in the Abacatal Quilombola community, aiming to assess species diversity in preserved forest environments. The region, home to approximately 400 inhabitants, is undergoing rapid urbanization, with deforestation and infrastructure projects altering local ecosystems [29]. These changes can impact mosquito populations, influencing their abundance, distribution, and potential epidemiological risks. Given the imminent risk of environmental degradation, entomofaunistic studies are essential to understanding the diversity of mosquito species, as they are vectors of arboviruses of epidemiological relevance [3]. Additionally, the area remains poorly explored in terms of biodiversity, reinforcing the urgency of detailed surveys. The findings may support more sustainable public policies that reconcile economic development with environmental conservation and social well-being.

## 2. Materials and Methods

### 2.1. Ethical Aspects

In order to carry out scientific activities involving the collection of mosquitoes in the Quilombola community of Abacatal, a request for authorization to carry out these activities was filed with the Evandro Chagas Institute, with the prior authorization of the community representatives, under internal record no. 3/2023. Likewise, authorization to collect biological samples was requested from the Biodiversity Authorization and Information System of the Chico Mendes Institute for Conservation and Biodiversity (SISBIO-ICMBio), under registration no. 82230-1.

### 2.2. Study Area

This study was carried out in the Quilombola community of Abacatal, a place that stands out for the preservation of a significant area of native vegetation and is characterized by a tropical climate, which exerts a significant influence on the local fauna. This climate is marked by high temperatures throughout the year and high humidity, creating ideal conditions for the development of mosquitoes [30,31].

The area is located in the rural area of the municipality of Ananindeua, 16 km from the capital Belém and 8 km from the urban center of Ananindeua, along the Alça Viária, in the state of Pará (01° 24′ 54.2″ S and 048° 20′ 22.2″ W), at an altitude of 27 m [29].

The remaining vegetation cover is predominantly made up of secondary forest, where large trees can be seen, with average heights varying between eight and ten meters. This vegetation not only enriches the local landscape, but also plays a fundamental role in maintaining biodiversity. There is usually frequent rainfall between November and April, which not only provides the necessary moisture for the lush vegetation, but also creates pools of water that become perfect breeding grounds for mosquitoes [32].

**Figure 1:**
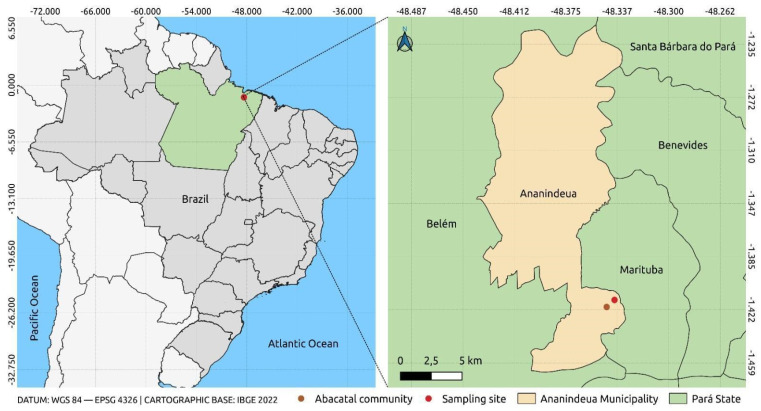
Location of the sample collection point. The figure was created using QGIS v.3.10.4 software together with publicly available georeferencing data from the Brazilian Institute of Geography and Statistics (IBGE), with satellite images from Google Earth v.10.77.0.1.

Geographically, the Abacatal Quilombola community is located 8 km from the urban center of Ananindeua, bounded by significant landmarks. To the east, it borders the Wildlife Reserve (REVIS), an important area for the preservation of local fauna and flora. To the west, it is bathed by the Una stream, and the community is also bordered by the Bom Jesus community. The area is subdivided into two physiographies: the terra firme zone and the insular region. This division not only influences the diversity of species present, but also the management and conservation practices implemented by the community (Figure 1) [33].

In short, the high temperatures and constant humidity create a favorable environment for the reproduction and survival of these insects. However, the tropical climate of the Quilombola community of Abacatal plays a crucial role in structuring the mosquito fauna. The interaction between climatic conditions, native vegetation, and the community’s management practices is fundamental to understanding the population dynamics of these insects and their influence on the health and well-being of the residents.

### 2.3. Collection Periods

Two collection campaigns were carried out, each lasting ten days, from Monday to Friday. The first collection was carried out in the rainy season, the specific sampling dates for both collection periods were from 20 April to 5 May in 2023, and the second campaign, lasting ten days, was in the drier period between 21 August and 1 September 2023. The collection methods used were Protected Human Attraction (PHA) and CDC-type light traps, following the recommendations of the Brazilian Ministry of Health [34].

#### 2.3.1. Protected and Enlightened Human Attraction

Recommended by the Evandro Chagas Institute and the Brazilian Ministry of Health [35], the Protected Human Attraction (PHA) method aims to collect adult female mosquitoes. The PHA technique was carried out by three professionals, one in the treetops (9 m) and two on the ground, in 3 h in the field. This technique involves collecting mosquitoes with diurnal habits, which are attracted by human odor, temperature, and sweat in search of blood. During the field collection expeditions, the mosquitoes were collected between 9 a.m. and 12 p.m. using an entomological hand net (puçá-type) and an oral aspirator.

#### 2.3.2. Collection with a CDC Trap

CDC-type light traps [36] without additional attractants were used to attract arthropods with positive phototaxis at night. Arthropods approach the trap, are immediately sucked into it by a fan, and are temporarily stored until collection by the team the following day. In this study, two traps were used: one positioned at the canopy level and the other at ground level, operating from 6 p.m. to 8 a.m. the next day, from Tuesday to Friday, resulting in an eight-day collection campaign.

#### 2.3.3. Taxonomic Identification

Taxonomic identification was based on observation of the external morphology of the specimens, using a chill table (Eletrohospitalar, Brasília, Distrito Federal, Brasil) set to approximately −30 °C, and Zeiss Stemi 2000-C stereomicroscopes (Carl Zeiss, Oberkochen, Germany). In addition, the dichotomous keys provided by Lane (1953) [37] and Forattini (2002) [31] were used to identify the specimens. The abbreviations for genus and subgenus followed the conventions proposed by Reinert (2009) [38]. After taxonomic identification, the specimens were grouped in batches (in 2 mL *Eppendorf* microtubes) containing individuals of the same species and were labeled with an internal registration number for arthropod samples.

### 2.4. Faunal Analysis

#### 2.4.1. Dominance of Species

Dominance categories were determined using the classification established by Friebe (1983) [39], where D% = (i/t) × 100, where i = total number of individuals of a species and t = total number of individuals collected. The categories are as follows: D > 10% = eudominant; D > 5% < 10% = dominant; D > 2% < 5% = subdominant; D = 1% < 2% = possible; and D < 1% = rare.

#### 2.4.2. Diversity Analysis

Diversity was estimated using Hill numbers [40,41], where q represents the order of diversity (qD), determining the relative influence of common and rare species in the estimation. The first three Hill numbers were evaluated: q = 0, which corresponds to species richness; q = 1, equivalent to the exponential of Shannon’s entropy index (H), which considers both species richness and evenness, giving greater weight to more abundant species; and q = 2, corresponding to the inverse of Simpson’s concentration index (D) reflects dominance and is inversely proportional to diversity, meaning that higher D values indicate lower diversity by increasing the probability that individuals belong to the same species. The indices were obtained using the R package hillR v.0.5.2 [42].

To estimate sampling effort and assess the sufficiency of sampling, the R iNEXT v.3.0.1 package was used to generate rarefaction and extrapolation curves based on species richness and individual abundance. Interpolation curves were constructed to compare the observed diversity between the analyzed periods, while extrapolation curves allowed for projecting the expected richness if the sampling effort was increased [41,43]. Input data included the abundance of individuals and the number of species identified in each period. The estimation method was based on the Chao1 model [44], which considers the frequency of rare species to adjust the projections. The analysis was performed with 1000 bootstrap iterations to ensure the robustness of the estimates and the reliability of the confidence intervals. The generated curves were interpreted to evaluate whether the sampling effort was sufficient to capture the actual diversity of the community or if additional sampling could reveal further species.

#### 2.4.3. Environmental Variables

The average temperature (°C) and relative humidity (%) were recorded every 60 min using a calibrated digital thermo-hygrometer. Precipitation data, accumulated over 24 h, were obtained from the National Institute of Meteorology (INMET) (https://portal.inmet.gov.br/) (acessed on 17 March 2025).

## 3. Results

A total of 3202 mosquito specimens were collected. Data are presented on the abundance and diversity of mosquitoes across two seasonal periods (rainy and dry), using the PHA and CDC light trap methods. The PHA method accounted for 1793 specimens, while the CDC method collected 1409 specimens. In total, the sampling effort amounted to 300 h, with 60 h dedicated to the PHA method and 240 h to the CDC method.

Of the total specimens collected, 1747 were found in the rainy season and 1455 in the dry season. A total of 56 mosquito species were identified across 13 genera. Abundance was higher during the rainy season, suggesting that this period is more favorable for mosquito survival and reproduction.

The most abundant genera were *Culex* Linnaeus, 1758, with 1519 individuals (47.44%), *Coquillettidia* Dyar, 1905, with 1061 individuals (33.14%), and *Cx.* (*Mel.*) *portesi* with 955 individuals, representing 29.83% of the total, showing its adaptability and the favorability of the environment. The method in which the greatest number was collected was CDC traps in the ground modality, and the species *Cq.* (*Rhy.*) *venezuelensis* was collected in greater abundance by human attraction in the ground, with 838 individuals, representing 26.17%.

The dominant species were *Cq.* (*Rhy.*) *arribalzagae* and *Cx.* (*Mel.*) *intricatus*, with a total of 203 and 291, respectively, while *An.* (*Ano.*) *mediopunctatus* is classified as rare, as shown in Table 1.

In terms of species composition, the two periods shared 38 taxonomic units, including epidemiologically significant species such as *Cx*. (*Mel*.) *portesi*, *Cq*. (*Rhy*.) *venezuelensis* and *Cq*. (*Rhy*.) *arribalzagae*. These species were highlighted as eudominant and dominant in the study, respectively. The diversity indices analyzed revealed differences between the rainy and dry seasons. During the rainy season, 1747 individuals were recorded, distributed across 56 taxonomic units, with a richness of 45 species. Shannon’s entropy index (H) was 14.03, indicating high diversity and evenness in species distribution, while the inverse of Simpson’s concentration index (D) was 5.02, suggesting lower dominance and greater heterogeneity within the community. In contrast, during the dry season, abundance decreased to 1455 individuals, with 38 taxonomic units and a richness of 29 species. Shannon’s entropy index (H) dropped to 7.13, reflecting a reduction in diversity and community evenness, while the inverse of Simpson’s concentration index (D) was 2.56, indicating increased dominance of certain species (Table 2).

**Figure 2:**
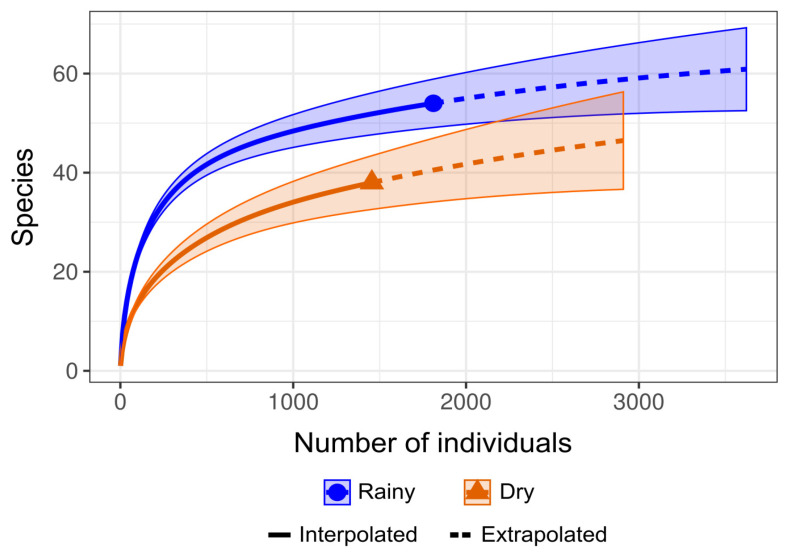
Graphical representation of diversity metrics based on species rarefaction curves of the number of individuals collected per species using the R iNEXT v.3.0.1 library, distributed over the research periods evaluated.

Regarding the sampling effort sufficiency estimates, the rarefaction and extrapolation curves generated for each study period exhibited a slight upward slope, indicating that a more intensive sampling effort could potentially reveal additional species (Figure 2). However, during taxonomic identification, some specimens could not be identified at the species level due to damage to their morphological structures. This limitation probably influenced the observed estimates, as the analysis considers both the number of species and the abundance of individuals sampled.

Regarding the environmental variables recorded during the study period (Supplementary Tables S1 and S2), relative humidity during the rainy period ranged from 78% to 89.5%, with an average of 82.4%. The average temperature varied from 23 °C to 28.35 °C, remaining below the values recorded in the dry period. Precipitation showed significant variation (0–13 mm), characterized by intermittent rainfall. In the dry period, relative humidity ranged from 41% to 79%, with an average of 69.1%. The temperature remained stable (27.35–29.85 °C), slightly higher than in the rainy period. Precipitation was extremely low, with only 3.8 mm recorded on a single day, reflecting the drought conditions typical of the Amazon summer.

## 4. Discussion

The Amazon region is recognized as one of the most important ecosystems in the world, due to its vast biological diversity and the complex network of interactions between its numerous species of plants and animals. Invertebrates, especially several species of Culicidae, play a crucial role in maintaining the ecosystem, as well as being vectors of pathogens that affect human and animal health [2].

The present study revealed a considerable diversity of Culicidae in the Quilombola community of Abacatal, with the identification of 56 species distributed across 13 genera. This finding aligns with previous research conducted in the metropolitan region of Belém, which includes the municipality of Ananindeua, and also highlights the richness and variability of Culicidae in different ecological contexts. Dias (2024) [19], while investigating a secondary forest fragment, identified 34 species of the subfamily Culicinae, distributed across 10 genera, with greater abundance and richness observed during the rainy season. Similarly, Farias (2019) [16], studying a protected area in an insular region, recorded 40 species distributed across 11 genera. The author observed higher abundance during the dry season but lower species richness, whereas during the rainy season, abundance was lower, but richness increased. These seasonal variations highlight the influence of climate and habitat type on mosquito population dynamics.

One of the most comprehensive studies on Culicidae diversity in the state of Pará was conducted by Xavier and Mattos (1975) [7], who sampled 307 sites across 64 municipalities, identifying 207 species belonging to 16 genera. This survey not only demonstrated the complexity of the mosquito fauna in the region but also became a reference for comparisons with contemporary studies, including the present work. The extensive geographic coverage and the recorded species richness reinforce the importance of the state of Pará as an area of high Culicidae diversity, corroborating the results obtained in this study.

Additionally, research conducted in other areas of the Amazon has also demonstrated similar diversity patterns. Along the Araçá River, in the northwestern Brazilian Amazon, 127 mosquito taxa were recorded, distributed across 17 genera, while along the Padauari River, in the state of Amazonas, 117 species were identified, also distributed across 17 genera [45,46]. Hendy et al. (2021) [47] conducted a study in a forest in Central Amazonia, Manaus, Amazonas, where they collected 2146 adult mosquitoes, representing seven genera and 34 species. Although species richness was lower compared to the present study, the high abundance of collected individuals reinforces the relevance of investigations in forested areas, which may harbor significant Culicidae populations with epidemiological potential.

In this study, the composition and distribution of mosquito species revealed a clear dominance pattern, with *Culex* being the most abundant and frequent genus. This pattern was primarily influenced by *Cx*. (*Mel.*) *portesi*, classified as eudominant, with 955 specimens collected, representing 29.83% of the total mosquitoes sampled. The genus *Coquillettidia* also exhibited high representativity, with *Cq. venezuelensis* as the most abundant species, accounting for 838 individuals, or 26.17% of the total sample. These findings align with those of Confalonieri and Costa-Neto (2012) [13]. A study conducted by Ramos (2022) [18] in the metropolitan region of Belém also observed the dominance of *Cx.* (*Mel.*) *portesi* and *Cq.* (*Rhy.*) *venezuelensis*, with collections carried out using PHA from 9 a.m. to 12 p.m. and CDC light traps. In addition to the dominant species mentioned, *Cq.* (*Rhy.*) *arribalzagae* and species from the Intricatus group of the *Culex* (*Melanoconion*) subgenus were frequently recorded, consistent with previous Amazonian studies [48]. Likewise, research conducted in the Amazon deforestation arc in northern Mato Grosso also identified *Cq.* (*Rhy.*) *arribalzagae* and *Culex* species as dominant.

The genus *Culex* holds significant epidemiological importance, as it is primarily involved in the transmission of various pathogens, including *Orthoflavivirus nilense* and *Orthoflavivirus japonicum*. Its remarkable ability to adapt to diverse environments poses a considerable challenge for vector control strategies [49]. In Brazil, studies have already detected a total of 42 arbovirus species in mosquitoes belonging to this genus, reinforcing its role as a key vector in the circulation of these viruses [50].

As already reported, *Cx.* (*Mel.*) *portesi* was among the species recorded in this study, reinforcing its known association with sylvatic environments and nocturnal activity. This mosquito exhibits a strong preference for feeding on small vertebrates and has been naturally infected with several medically relevant arboviruses. Among them is *Alphavirus venezuelan*, isolated in Venezuela [51], while multiple arboviruses have been detected in different locations within the state of Pará, Brazil, including *Alphavirus mucambo*, *Hapavirus mosqueiro*, *Orthobunyavirus maritubaense* [52], Bussuquara virus, *Orthoflavivirus ilheusense* [15], and *Orthoflavivirus louisense* [53]. Its presence highlights the need for ongoing surveillance, especially in areas where human populations interact with forested habitats.

Specimens of *Culex (Melanoconion) vomerifer* were also recorded, all exclusively collected in the dry period using CDC traps at the ground level. However, previous studies have reported females engaged in blood-feeding activity both at ground level and in the canopy in habitats such as forest edges, regenerating secondary vegetation, partially deforested forest areas, as well as environments near mangroves and wetlands [54,55,56]. This species has been found naturally infected with *Orthobunyavirus caraparuense*, *Orthobunyavirus oribocaense*, *Orthobunyavirus guamaense* [52], and *Orthobunyavirus ananindeuense* [57,58]. These infections have been recorded in the state of Pará, reinforcing the epidemiological significance of this species and the necessity for continuous surveillance in the region.

The genus *Coquillettidia* is widely studied and distributed across tropical and subtropical regions, particularly in the Americas, Asia, and Africa. Some species within this genus are known for their aggressive biting behavior [31,59]. Among them, *Cq.* (*Rhy.*) *venezuelensis* stands out due to its highly aggressive nature [31] and its medical significance, as it has been found naturally infected with various arboviruses. These include *Orthoflavivirus nilense* [60,61], *Arurhavirus aruac*, Bimiti virus [62], *Orthobunyavirus gamboaense* [63], *Alphavirus mayaro* [64,65], *Phlebovirus itaporangaense*, Bussuquara virus, and Moju virus [66]. Furthermore, *Cq. venezuelensis* is considered a secondary vector of *Orthobunyavirus oropouchense*, reinforcing its potential epidemiological importance [67,68,69,70,71].

In the present study, *Cq.* (*Rhy.*) *arribalzagae* stood out as the dominant species, with 203 specimens collected, representing 6.34% of the total mosquitoes sampled. This predominance may be associated with its adaptability to different landscape gradients, as evidenced by previous studies [72,73]. For example, Vieira et al. (2021) [74], in a study conducted in Southern Amazonia, investigated mosquito diversity along a deforestation gradient, highlighting the occurrence of *Cq. arribalzagae* in both preserved and disturbed environments. These findings reinforce the hypothesis that the species exhibits ecological plasticity, allowing it to colonize habitats with varying levels of anthropogenic intervention.

Despite the scarcity of information regarding its vector competence, *Cq. arribalzagae* has been naturally found infected with *Orthobunyavirus oribocaense*, *Alphavirus una*, and *Orthobunyavirus wyeomyiae* [66,75]. This suggests a potential role for the species in the transmission of arboviruses, although further studies are needed to elucidate its vector competence and implications for public health. Additionally, the species exhibits both diurnal and nocturnal activity along with anthropophilic behavior characteristics that may favor its interaction with humans and, consequently, increase the risk of pathogen transmission.

In this study, as already observed, a wide diversity of species with recognized epidemiological importance was recorded. Within the genus *Aedes* Meigen, 1818, five species were identified, among which *Aedes albopictus*, known as the “Asian tiger mosquito”, stands out as a relevant vector of the *Alphavirus chikungunya* and other arboviruses. Damasceno-Caldeira et al. (2023) [76] conducted experimental studies that also identified this species as a potential vector of *Orthoflavivirus flavi* [77]. 

Specimens of *Aedes* (*Ochlerotatus*) *serratus* were also recorded. This species is characterized as a synanthropic mosquito frequently found in anthropized environments and subject to environmental impacts [31,78]. Additionally, it has been naturally detected as a carrier of several arboviruses, including *Orthoflavivirus louisense*, *Orthoflavivirus ilheusense*, *Oropouche orthobunyavirus*, *Orthobunyavirus caraparuense* [22,23,24], *Alphavirus aura* [77], and *Alphavirus venezuelan* [66]. Infections with *Alphavirus una*, *Orthobunyavirus oribocaense*, *Alphavirus mucambo*, and *Orthobunyavirus mirimense* [52,66] have been recorded.

Another mosquito species of epidemiological relevance recorded in this study was *Haemagogus janthinomys*. This species plays an important role in the maintenance and transmission of arboviruses in sylvatic environments, particularly among non-human primates, acting as a primary vector in forested areas. Notably, *Hg. janthinomys* is a well-documented vector of *Orthoflavivirus flavi* and *Alphavirus mayaro* [67,79,80,81], viruses of significant public health concern, especially in tropical forest regions. While its transmission cycle is predominantly restricted to wild habitats [24], the potential for spillover to human populations underscores the importance of continuous vector surveillance [82].

Genera belonging to the tribe Sabethini Blanchard, 1905, such as *Sabethes* Robineau-Desvoidy, 1827, *Limatus* Theobald, 1901, and *Wyeomyia* Theobald, 1901, were recorded. Within the genus *Sabethes*, eight species were identified, highlighting the diversity and distribution of this group in the studied region. Among the species, *Sabethes* (*Sabethoides*) *chloropterus* and *Sabethes* (*Sabethoides*) *glaucodaemon* stand out, both recognized for their role in the sylvatic *Orthoflavivirus flavi* transmission cycle [23]. These mosquitoes are predominantly sylvatic, typically inhabiting the forest canopy, commonly found in forested habitats, where they use phytotelmata such as tree holes, bromeliads, and bamboo internodes for larval development [13,31]. Additionally, some *Sabethes* species have demonstrated the ability to adapt to human-modified environments [83], and exhibit a primatophilic feeding habit, meaning they preferentially feed on non-human primates but will also bite humans when available [84]. This behavior increases the risk of *Orthoflavivirus flavi* transmission in areas where humans and primates coexist.

Regarding the genus *Limatus*, two species were identified: *Limatus* (*Limatus*) *flavisetosus* and *Limatus* (*Limatus*) *durhamii*. *Li. flavisetosus* is commonly observed in environments with a certain degree of landscape preservation, developing in phytotelmata such as bamboo internodes and coconut husks, although it can also adapt to artificial breeding sites [85]. This species has been naturally found infected with the *Orthobunyavirus maguariense* [66]. In contrast, *Li. durhamii* stands out for its high adaptability to urbanized environments, with studies even suggesting its potential for domiciliation [86]. This species has been recorded in areas with various levels of anthropization [87,88,89], highlighting its ecological versatility. Due to its anthropophilic behavior, *Li. durhamii* has a high potential to act as a vector of arboviruses, having been naturally found infected with the *Orthobunyavirus maguariense*, Tucunduba virus, *Orthobunyavirus wyeomyiae,* and *Gamboa orthobunyavirus* [66]. In a study conducted by Barrio-Nuevo et al. (2020) [90], *Orthoflavivirus zikaense* was detected in *Li. durhamii*. However, further experimental studies are needed to elucidate this species’ capacity to act as an efficient vector of these arboviruses.

In this study, the genus *Wyeomyia* was represented by 367 specimens, accounting for 11.45% of the total mosquitoes sampled. Ten species were identified, among which *Wyeomyia argenteorostris* was the most dominant, with 114 individuals recorded, representing 3.56% of the total specimens collected. Species of the genus *Wyeomyia* are primarily characterized as mosquitoes with a predominantly sylvatic distribution. However, records of their occurrence in forest fragments with varying levels of anthropogenic disturbance have been documented in previous studies [19,91,92,93]. These findings highlight the remarkable adaptability of these species to environments altered by human activity. Although the genus is generally considered to have little or no epidemiological relevance in the transmission of infectious and parasitic agents, some species have been found naturally infected with arboviruses. Among them, *Wy.* (*Tra*.) *aporonoma* and *Wy.* (*Den.*) *ypsipola* were found naturally infected with *Orthobunyavirus insulae*, while *Wy.* (*Tra.*) *aporonoma* was also associated with *Orthobunyavirus wyeomyiae* [66]. These records suggest that, although the epidemiological role of the *Wyeomyia* genus is not yet fully understood, certain species may contribute to the circulation of arboviruses in specific ecological contexts.

This study reveals the remarkable diversity of mosquitoes in the investigated area and emphasizes the urgent need for research focused on the identification and mapping of Culicidae fauna in the Amazon, particularly in urbanized areas facing intense environmental pressures, such as the Quilombola community of Abacatal. The accelerated urbanization process and its resulting transformations can significantly alter the bioecological and behavioral aspects of mosquito species, modifying pathogen transmission dynamics and, consequently, posing new public health challenges [94,95,96]. In this context, it becomes essential to understand how these environmental transformations influence population parameters, such as density, and ecological characteristics, such as diversity, abundance, and species dominance, as determining factors associated with the risk of disease transmission.

## 5. Conclusions

The present study assessed the mosquito fauna in the Quilombola community of Abacatal, recording 56 species from 13 genera, corroborating and expanding previous findings in the metropolitan region of Belém. The rainy season showed greater abundance and species diversity compared to the dry season, with *Cx.* (*Mel.*) *portesi* and *Cq.* (*Rhy.*) *venezuelensis* standing out as eudominant species.

The PHA captured more Cq. (Rhy.) *venezuelensis*, while CDC light traps recorded a higher number of *Cx.* (*Mel.*) *portesi*, highlighting the importance of complementary methods, such as the Shannon trap, for more representative sampling. Mosquito diversity was higher during the rainy season, with greater uniformity and heterogeneity, while the dry season exhibited lower diversity and higher species dominance. Rarefaction analyses suggest that additional collection efforts could expand species records, although taxonomic limitations due to morphological damage may have affected richness estimates.

The presence of epidemiologically relevant species underscores the need for continuous monitoring to assess public health risks. Future studies should include viral isolation techniques and metagenomic sequencing to better understand mosquito–virus interactions, enabling the early identification of pathogens, and allowing the development of effective prevention and control strategies to reduce risks to both human and animal populations.

## Figures and Tables

**Table 1:** Culicidae species collected during the rainy and dry season by PHA and CDC methods, both on the ground and in the canopy.

Species	Rainy				Dry					
	PHA		CDC		PHA		CDC			
	Ground	Canopy	Ground	Canopy	Ground	Canopy	Ground	Canopy	Total	Fre%
*Ae.* (*How.*) *fulvithorax*	6	0	0	0	3	0	0	0	9	0.28
*Ae.* (*Och.*) *fulvus*	0	0	0	0	1	0	0	0	1	0.03
*Ae.* (*Och.*) *scapularis*	1	1	0	0	0	0	0	0	2	0.06
*Ae.* (*Och.*) *serratus*	8	0	1	0	18	0	1	0	28	0.87
*Ae.* (*Pro.*) *argyrothorax*	9	0	0	0	0	0	0	0	9	0.28
*Ae.* (*Stg.*) *albopictus*	0	0	1	0	0	0	0	0	1	0.03
*An.* (*Ano.*) *mediopunctatus*	0	0	0	0	0	0	0	1	1	0.03
*An.* (*Ano.*) sp.	0	0	0	0	1	0	0	1	2	0.06
*An.* (*Ste.*) *nimbus*	1	0	0	0	0	0	0	0	1	0.03
*Cq.* (*Rhy.*) *arribalzagae*	198	0	1	0	0	0	2	2	203	6.34
*Cq.* (*Rhy.*) *nigricans*	8	0	0	0	1	0	0	0	9	0.28
*Cq.* (*Rhy.*) sp.	11	0	0	0	0	0	0	0	11	0.34
*Cq.* (*Rhy.*) *venezuelensis*	491	1	51	5	221	1	59	9	838	26.17
*Cx.* (*Car.*) *urichii/anduzei*	0	0	1	0	0	0	0	0	1	0.03
*Cx.* (*Cux.*) *declarator*	0	0	0	0	0	0	1	0	1	0.03
*Cx.* (*Cux.*) sp.	0	0	4	1	1	0	0	0	6	0.19
*Cx.* (*Mel.*) *faurani*	19	0	0	0	0	0	0	0	19	0.59
*Cx.* (*Mel.*) *gnomatos*	0	0	8	0	0	0	0	0	8	0.25
*Cx.* (*Mel.*) Atratus group	0	0	1	0	0	2	0	1	4	0.12
*Cx.* (*Mel.*) Intricatus group	180	0	76	35	0	0	0	0	291	9.09
*Cx.* (*Mel.*) *pedroi*	0	0	14	0	1	0	4	0	19	0.59
*Cx.* (*Mel.*) *portesi*	19	0	61	1	19	0	834	21	955	29.83
*Cx.* (*Mel.*) sp.	11	0	13	33	5	0	31	25	118	3.69
*Cx.* (*Mel.*) *spissipes*	2	0	23	0	1	0	33	0	59	1.84
*Cx.* (*Mel.*) *vomerifer*	0	0	0	0	0	0	38	0	38	1.19
*Hg.* (*Hag.*) *janthinomys*	4	27	0	1	2	2	0	0	36	1.12
*Jn. longipes*	1	0	0	0	0	0	0	0	1	0.03
*Li. durhamii*	5	0	1	0	3	0	0	0	9	0.28
*Li. flavisetosus*	10	0	0	0	6	0	0	0	16	0.50
*Limatus* sp.	5	0	0	0	0	0	0	0	5	0.16
*Ma.* (*Man.*) *indubitans*	1	0	0	0	1	0	0	0	2	0.06
*Ma.* (*Man.*) *pseudotitillans*	0	0	2	1	0	0	0	0	3	0.09
*Ma.* (*Man.*) sp.	0	0	0	1	0	0	0	0	1	0.03
*Ma.* (*Man.*) *titillans*	0	0	0	0	0	1	0	0	1	0.03
*Ps.* (*Jan.*) *albipes*	0	0	0	0	1	1	0	0	2	0.06
*Ps.* (*Jan.*) *ferox*	4	1	0	0	0	0	0	0	5	0.16
*Sa.* (*Sab.*) *tarsopus*	0	2	0	0	0	0	0	0	2	0.06
*Sa.* (*Sab.*) *amazonicus*	0	7	0	0	0	0	0	0	7	0.22
*Sa.* (*Sab.*) *belisarioi*	0	15	0	0	1	2	0	0	18	0.56
*Sa.* (*Sab.*) *cyaneus*	0	7	0	0	3	0	0	0	10	0.31
*Sa.* (*Sab.*) *foratinii*	0	0	0	0	1	0	0	0	1	0.03
*Sa.* (*Sab.*) *quasicyaneus*	0	1	0	0	2	0	0	0	3	0.09
*Sa.* (*Sbo.*) *chloropterus*	0	12	0	0	0	11	0	0	23	0.72
*Sa.* (*Sbo.*) *glaucodaemon*	0	13	0	0	1	2	0	0	16	0.50
*Sabethes* sp.	0	1	0	0	0	0	0	0	1	0.03
*Tr.* (*Trc.*) *digitatum*	5	0	0	0	0	0	0	0	5	0.16
*Ur.* (*Ura.*) *geometrica*	0	0	5	0	0	3	0	1	9	0.28
*Ur.* (*Ura.*) *hystera*	0	0	0	0	0	11	0	2	13	0.41
*Ur.* (*Ura.*) *natalie*	0	0	0	0	0	9	0	0	9	0.28
*Uranotaenia* (*Ura.*) sp.	0	0	2	0	0	1	0	0	3	0.09
*Wy.* (*Cru.*) *dyari*	0	0	0	0	1	0	0	0	1	0.03
*Wy.* (*Den.*) *complosa*	12	0	0	0	0	0	0	0	12	0.37
*Wy.* (*Den.*) *luteoventralis*	30	0	0	0	0	0	0	0	30	0.94
*Wy.* (*Den.*) *ypsipola*	3	0	0	0	0	0	0	0	3	0.09
*Wy.* (*Pho.*) sp.	1	0	0	0	0	0	0	0	1	0.03
*Wy.* (*Pho.*) *splendida*	2	0	0	0	0	0	0	0	2	0.06
*Wy.* (*Tra.*) *aporonoma*	29	0	0	0	1	0	0	0	30	0.94
*Wy.* (*Tra.*) *staminifera*	6	0	0	0	0	0	0	0	6	0.19
*Wy.* (*Wyo.*) *hemisagnosta*	3	3	0	0	0	0	0	0	6	0.19
*Wy. argenteorostris*	88	2	0	0	24	0	0	0	114	3.56
*Wy. flui*	7	0	0	0	5	0	0	0	12	0.37
*Wy. negrensis/occulta*	38	0	0	0	4	0	0	0	42	1.31
*Wyeomyia* sp.	92	1	0	0	15	0	0	0	108	3.37
Abundance	1747				1455				3202	100.00

**Legend:** freq. % = D > 10% eudominant; D > 5 < 10% dominant; D > 2% < 5% subdominant; D = 1 < 2% possible; and D <1% rare; PHA (Protected Human Attraction) and CDC (Centers for Disease Control and Prevention).

**Table 2:** Description of the diversity indices.

	Rainy	Dry
**Abundance**	1747	1455
**Taxonomic units**	56	38
**Richness**	45	29
**Shannon’s entropy index (H)**	14.03	7.13
**Inverse of Simpson’s concentration index (D)**	5.02	2.56

## Data Availability

The original contributions presented in this study are included in the article/Supplementary Material. Further inquiries can be directed to the corresponding author.

## Supplementary Materials

The following supporting information can be downloaded at https://www.mdpi.com/article/10.3390/insects16040397/s1, Supplementary Table S1. Meteorological variables recorded during the rainy period, with values representing the daily averages of relative humidity (%), temperature (°C), and precipitation (mm). Supplementary Table S2. Meteorological variables recorded during the dry period, with values representing the daily averages of relative humidity (%), temperature (°C), and precipitation (mm).
