# Supplementary material for: Culicidae Fauna (Diptera: Culicomorpha) of the Quilombola Community of Abacatal, Ananindeua, Pará, in the Brazilian Amazon
Source: Insects. 2025 Apr 10;16(4):397. doi: 10.3390/insects16040397 (PMC12027505; doi:10.3390/insects16040397)
Supplement: Supplementary file 1 [file insects-16-00397-s001.zip › insects-3511956-supplementary.pdf]

Supplementary Table S1. Meteorological variables recorded during the rainy period, with values representing the daily averages of relative humidity (%), temperature (°C), and precipitation (mm).

Rainy			
**Date**	**Humidity (%)**	**Temperature (C°)**	**Precipitation (mm)**
20/04/2023	89	28.35	3
24/04/2023	87.5	26.65	0.2
25/04/2023	89.5	27.2	3.6
26/04/2023	79.5	24.75	0
27/04/2023	79.5	24.75	3.6
28/04/2023	78	23.15	13
02/05/2023	79.5	23	7.6
03/05/2023	79.5	27.25	0
04/05/2023	84	26.4	0
05/05/2023	85	25.95	7.4

Supplementary Table S2. Meteorological variables recorded during the dry period, with values representing the daily averages of relative humidity (%), temperature (°C), and precipitation (mm).

Dry			
**Date**	**Humidity (%)**	**Temperature (C°)**	**Precipitation (mm)**
21/08/2023	73	28.75	0
22/08/2023	41	29.85	3.8
23/08/2023	69	28.9	0
24/08/2023	70.5	28.45	0
25/08/2023	70.5	28.45	0
28/08/2023	70.5	27.65	0
29/08/2023	79	27.6	0
30/08/2023	68.5	28.9	0
31/08/2023	73	28.25	0
01/09/2023	75.5	27.35	0
